# Rice Responses to Abiotic Stress: Key Proteins and Molecular Mechanisms

**DOI:** 10.3390/ijms26030896

**Published:** 2025-01-22

**Authors:** Xiaohui Wang, Xuelei Liu, Yonglin Su, Huaizong Shen

**Affiliations:** 1 Key Laboratory of Systems Health Science of Zhejiang Province, School of Life Science, Hangzhou Institute for Advanced Study, University of Chinese Academy of Sciences, Hangzhou 310024, China; suyonglin23@mails.ucas.ac.cn; 2 Zhejiang Key Laboratory of Structural Biology, School of Life Sciences, Westlake University, Hangzhou 310024, China; liuxuelei@westlake.edu.cn; 3 Westlake Laboratory of Life Sciences and Biomedicine, Hangzhou 310024, China

**Keywords:** abiotic stress, molecular mechanism, rice, stress resistance, breeding

## Abstract

The intensification of global climate change and industrialization has exacerbated abiotic stresses on crops, particularly rice, posing significant threats to food security and human health. The mechanisms by which rice responds to these stresses are complex and interrelated. This review aims to provide a comprehensive understanding of the molecular mechanisms underlying rice’s response to various abiotic stresses, including drought, salinity, extreme temperatures, and heavy metal pollution. We emphasize the molecular mechanisms and structural roles of key proteins involved in these stress responses, such as the roles of SLAC1 and QUAC1 in stomatal regulation, HKT and SOS proteins in salinity stress, heat shock proteins (HSPs) and heat stress transcription factors (HSFs) in temperature stress, and Nramp and ZIP transport proteins in response to heavy metal stress. This review elucidates the complex response networks of rice to various abiotic stresses, highlighting the key proteins and their related molecular mechanisms, which may further help to improve the strategies of molecular breeding.

## 1. Introduction

Rice serves as a staple food for a significant portion of the global population and is highly sensitive to environmental changes. It is susceptible to various abiotic stresses, including drought, salinity, extreme temperatures, and heavy metal pollution [1]. These stresses severely impact the growth and development of rice, posing direct threats to food security and human health. As the global population continues to grow, it is projected to reach between 9.7 and 10 billion by 2050, leading to a substantial increase in food demand. Concurrently, climate change is exacerbating water scarcity, presenting serious challenges to agricultural production [2]. As one of the world’s major staple crops, the yield and stability of rice are crucial for ensuring global food security. Therefore, understanding the mechanisms by which rice responds to these abiotic stresses is essential for developing stress-resistant varieties and enhancing crop yields.

Drought stress is particularly challenging for rice cultivation, which requires substantial water. In conditions of water scarcity, rice mitigates damage by regulating stomatal movement, ensuring membrane stability, and accumulating osmotic protectants like soluble sugars and proline to maintain turgor pressure [3]. Drought also elevates oxidative stress, generating reactive oxygen species (ROS) that can damage cell membranes and organelles. Salt stress further complicates rice cultivation [4].

Salt stress is another major obstacle in rice cultivation. High salinity reduces soil water potential, leading to osmotic stress and limiting water uptake. This stress is compounded by excessive sodium (Na^+^) disrupting potassium (K^+^) absorption, resulting in ionic stress [5]. Rice counters these effects by accumulating organic solutes such as proline and betaine, and employing HKT family proteins and the SOS signal pathway to maintain K^+^/Na^+^ homeostasis.

Temperature extremes, both high and low, significantly affect rice growth and development. Low temperatures can impede growth, while high temperatures can cause protein denaturation and cellular damage. Rice responds by activating molecular mechanisms that include modifications in cell wall and membrane components, changes in membrane protein conformation, increases in cytosolic calcium, elevated ROS levels, and the expression of temperature-responsive genes [6].

Heavy metal stress, particularly from cadmium (Cd) and arsenic (As), severely impacts rice growth and threatens food safety [7]. Rice mitigates heavy metal toxicity by regulating the absorption and transport of these metals and utilizing metal chelators such as metallothioneins (MTs) and phytochelatins (OsPCS).

**Figure 1:**
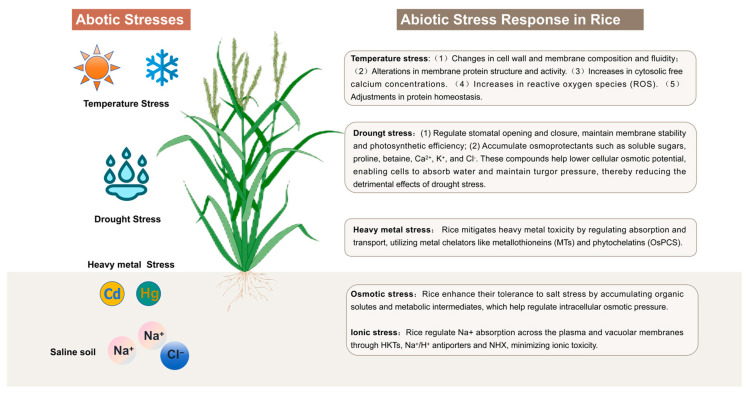
Overview of rice responses to abiotic stresses. Multiple physiological responses of rice under different abiotic stresses, including temperature, drought, heavy metals, osmotic stress, and ionic stress, are illustrated. (The rice plant icon is created with BioRender.com).

Understanding rice’s responses to these abiotic stresses involves multiple dimensions, including stress perception, signal transduction, and transcriptional regulation. Despite the availability of comprehensive reviews on this topic [8,9,10,11,12], our study distinguishes itself through several novel contributions. First, we present an in-depth molecular-level analysis of key proteins involved in rice’s response to abiotic stresses, including the functional roles of SLAC1 and QUAC1 in stomatal regulation, HKT and SOS proteins in salinity stress, heat shock proteins (HSPs) and heat stress transcription factors (HSFs) in temperature stress, and Nramp and ZIP transport proteins in heavy metal stress responses. Second, we highlight the structural characteristics of these proteins and their critical amino acid sites, which represent potential targets for molecular breeding strategies. Third, we systematically explore the intricate response networks of rice to various abiotic stresses (as summarized in Figure 1), elucidating the shared regulatory mechanisms underlying different stress responses. By unraveling these complex networks, our review identifies key molecular targets for developing stress-resistant rice varieties, offering critical insights for improving crop yields and ensuring global food security.

## 2. Drought Stress

Drought is one of the major abiotic stresses faced by global agriculture, significantly impacting rice, an important staple crop [2,13]. Rice is particularly susceptible to drought due to its small root system, thin cuticle, and rapid stomatal closure. Water stress is a critical factor limiting plant growth, affecting not only photosynthesis and respiration but also directly influencing cell expansion and division [14]. Insufficient water supply not only leads to a decline in cellular water potential, triggering dehydration and impairing cellular metabolism, but also increases oxidative stress levels, resulting in the production of ROS that disrupt the normal redox balance. This oxidative damage affects photosynthetic pigments, membrane lipids, proteins, and nucleic acids, ultimately hindering plant growth or leading to death [15].

The balance between growth and survival is especially important under moderate and recurrent water stress conditions [10,11]. Drought-resistant plants or cultivars need to reduce above-ground growth to minimize water evaporation while maintaining or increasing root growth to enhance water absorption capacity [10]. For example, TOR signaling represses ABA signaling and stress responses in unstressed conditions, whereas ABA signaling represses TOR signaling and growth during times of stress. Plants utilize this conserved phospho-regulatory feedback mechanism to optimize the balance of growth and stress responses [16].

To cope with drought stress, plants have evolved a series of physiological adaptive mechanisms. Under drought conditions, plants typically close their stomata to reduce water loss. While this helps conserve water, it also leads to reduced carbon dioxide uptake, thereby inhibiting photosynthesis. Furthermore, drought stress promotes the synthesis of osmotic adjustment substances, such as proline and sugars, which help maintain cell turgor and protect cellular structures. The accumulation of these metabolites is a key response for plant survival under prolonged water scarcity [17].

Rice exhibits a distinct phase-specific response to drought stress, characterized by temporal lag effects [18]. The response mechanisms and affected physiological processes vary at different growth stages, with the reproductive stage being the most significantly impacted. Water scarcity disrupts floral bud differentiation, pollination, and fertilization, leading to an increase in empty grains and insufficient grain filling, ultimately resulting in a substantial decline in yield and quality [19]. The transmission of drought signals and the regulation of gene expression also exhibit temporal delays. For instance, drought stress initially triggers a rapid response through the abscisic acid (ABA) signaling pathway, such as stomatal closure to reduce water transpiration. Subsequently, the DREB (Drought-Responsive Element-Binding) pathway elicits a delayed response that enhances the plant’s long-term drought tolerance, albeit with adverse effects on growth. This phased and time-lagged response strategy enables rice to adapt flexibly to the ever-changing drought environment [18].

Recent studies have identified several key genes and proteins that play important roles in the drought response of rice at the molecular level. Overexpression of *OsDof12* can upregulate genes associated with the phenylalanine metabolic pathway, increase the accumulation of phenolic compounds, reduce reactive oxygen species (ROS) damage, and interact with OsGI, revealing a novel regulatory mechanism under drought conditions [20]. OsMRLK63, an MRLK protein located on the plasma membrane, interacts with and phosphorylates NADPH oxidases such as OsRbohA, OsRbohB, and OsRbohH, thereby regulating ROS production and enhancing drought tolerance in rice [21]. OsERF103 has been identified as a key regulator of drought-responsive gene expression in rice, demonstrating its cooperative interaction with SNAC1 and recruitment of OsSDG705, which influences the H3K4me3 levels at the *OsbZIP23* locus and other drought-related gene loci [22]. These findings provide a potential target for genetic engineering and breeding of drought-tolerant rice varieties. OsPUB75–OsHDA716 negatively regulates drought tolerance in rice by mediating the deactivation and degradation of OsbZIP46 [23]. A genome-wide association study (GWAS) identified the *ROOT LENGTH 1 (RoLe1)* locus on chromosome 1, which regulates root development and drought resistance in rice. The transcription of *RoLe1* is directly regulated by OsNAC41, and RoLe1 interacts with OsAGAP, altering auxin transport (PAT) in roots, thereby controlling root length and drought resistance [24]. ONAC023 has been identified as a central regulatory factor in rice’s response to drought and heat stress, conferring tolerance during both vegetative and reproductive stages. Drought and heat stress induce the abundance of *ONAC023* transcripts and facilitate its nuclear translocation with the assistance of the remorin protein OsREM1.5. ONAC023 regulates various genes involved in redox homeostasis, water transport, and alternative splicing, revealing the multi-layered regulation of NAC, which provides valuable genetic resources for breeding drought- and heat-tolerant rice [25].

In the process of adapting to drought environments, upland rice and lowland rice exhibit distinct morphological and physiological traits. Upland rice typically possesses a more developed root system and thicker leaf cuticles, which enhance water absorption and reduce transpiration loss [26]. These morphological adaptations contribute to improved water use efficiency and survival capability of the plants under drought conditions. Next, we will delve into the physiological responses of rice under drought stress, particularly focusing on the ion channels associated with stomatal closure and their regulatory mechanisms, as well as the role of receptor proteins involved in osmotic regulation.

### 2.1. Ion Channels Mediating Stomatal Closure in Response to Drought Stress

Changes in the turgor pressure of guard cells are crucial for regulating stomatal movement, with anion channel activation playing a key role in stomatal closure. There are two types of anion channels in guard cells: the Slow Anion Channel (SLAC), which responds slowly to membrane voltage changes and primarily conducts Cl^−^ and NO_3_^−^, and the Quick Anion Channel (QUAC), which activates within milliseconds and mainly conducts malate ions.

**Figure 2:**
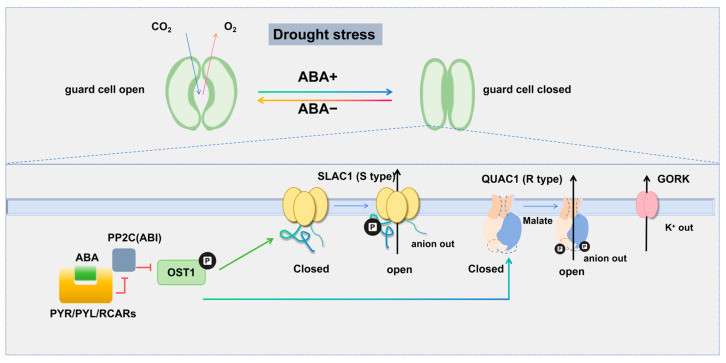
Regulation of the stomatal closure mechanism in plants under drought stress. Under normal conditions, stomata are open, allowing for the exchange of carbon dioxide (CO_2_) and oxygen (O_2_). When plants sense drought stress, the level of abscisic acid (ABA) increases. ABA binds to the PYR/PYL/RCAR receptors, inhibiting PP2C (ABI) and activating OST1. OST1 phosphorylates SLAC1, transitioning it from an inactive to an active state, which leads to the efflux of anions from guard cells, resulting in membrane depolarization. To balance the electrochemical potential, K^+^ exit through outward-rectifying potassium channels (GORK). The efflux of K^+^ decreases the internal osmotic pressure of guard cells, causing further water loss from guard cells, which leads to a decrease in turgor pressure, resulting in guard cell shrinkage and stomatal closure. The activity of QUAC1 can be regulated by malate and phosphorylation by the plant kinase OST1. QUAC1 works in concert with SLAC1 to regulate stomatal closure. PYR/PYL/RCAR—PYR (Pyrabactin Resistance), PYL (PYR-like), RCAR (Regulatory Component of ABA Receptor): A group of receptors that can bind to ABA and regulate plant responses to drought stress. PP2C—Protein Phosphatase 2C; OST1—Open Stomata 1; SLAC1—Slow Anion Channel 1; GORK—Outward Rectifying Potassium Channel; QUAC1—Quick Anion Channel 1.

Under drought stress, a critical plant response is the synthesis of abscisic acid (ABA) [27,28]. In *Arabidopsis thaliana*, the core ABA signaling pathway has been relatively well identified [29]. Increased ABA levels compete with its receptor PYLs for the phosphatase ABI1, forming the ABA-PYL-ABI1 ternary complex. This leads to the dissociation of the kinase OST1 from the OST1-ABI1 complex, resulting in the phosphorylation and activation of the downstream ion channel SLAC1 [30,31]. Activated SLAC1 facilitates anion efflux from guard cells, causing membrane depolarization. To restore electrochemical balance, K^+^ ions exit through outward potassium channels (GORK), leading to reduced internal osmotic pressure, further water loss from guard cells, decreased turgor pressure, and ultimately stomatal closure [32,33] (Figure 2). The ABA signaling mechanism in rice is similar to that in Arabidopsis in terms of core components and modes of action [34]. QUAC1 (also known as ALMT12) is another critical quick anion channel that mediates stomatal closure [35]. It is activated by malate and exhibits specific voltage dependence, with Ca^2+^ and calmodulin (CaM) regulating its activity [36]. Single mutants of SLAC1 and QUAC1/ALMT12 retain partial responses to ABA, darkness, reduced air humidity, and elevated CO_2_, while double mutants completely lose responsiveness, indicating that these channels work together to control stomatal closure [37]. In rice, OsASR1 is vital for ABA-mediated stomatal closure under drought stress, while OsASR1 and OsASR5 from upland rice enhance drought tolerance through ABA and H_2_O_2_ signaling pathways, improving crop yields [38].

OsSLAC1 is a nitrate-selective anion channel with limited permeability to chloride, malate, or sulfate [39,40]. Its expression in the *Arabidopsis slac1-3* mutant rescued the hypersensitive drought phenotype. Although research on SLAC1 in rice is limited and its three-dimensional structure remains unknown, sequence alignment shows significant homology with AtSLAC1 [41]. AtSLAC1 is a trimeric protein with ten transmembrane (TM) helices in each monomer, forming a central pore. In its resting state, the channel is inactive due to a narrow pore diameter and a “plug-like” structure blocking the intracellular region. N-terminal region phosphorylation by upstream kinases induces a conformational change, releasing the “plug” and allowing pore dilation for anion passage, thereby influencing turgor pressure and stomatal movement [42,43] (Figure 2).

The *aluminum-activated malate transporter* (*ALMT*) gene family in rice comprises nine members (*OsALMT1-9*). Increased expression of OsALMT4 enhances malate efflux, raising its concentration in the extracellular matrix, including the xylem, which may disrupt nutrient transport and affect manganese distribution in tissues [44]. OsALMT7 may mediate malate influx or efflux in spikelet cells based on membrane potential and malate concentrations [45]. Currently, no structural information is available for ALMT family members from rice, but the three-dimensional structure of GwQUAC1 provides insights into its gating mechanism. GwQUAC1 is a symmetric homodimer with six TMs per subunit. The interaction between subunits creates a twisted bilayer structure with a T-shaped central pore in a high-energy state. Electrophysiological analyses indicate that GwQUAC1 has rapid activation and inactivation kinetics, likely due to its high-energy state, allowing frequent transitions between open and closed states. When QUAC1 binds to malate, a conformational change occurs on TM4, promoting the transition from a closed to an open state. Additionally, QUAC1 activation is not only directly activated by malate, but also dependent on phosphorylation or calmodulin. This diverse activation mechanism enables the channel to respond quickly to external stress, facilitating stomatal closure regulation [46].

### 2.2. Drought Stress Receptor—OSCA1

Osmotic regulation is vital for plants to mitigate drought-induced damage and maintain physiological functions. In water-deficient environments, plants trigger high osmotic stress signals that increase cytosolic free calcium ion concentration ([Ca^2+^]i), known as hyperosmolality-induced [Ca^2+^]i increase (OICI). By elevating solute concentration, plants can reduce osmotic potential, prevent excessive water loss, and maintain turgor pressure, ensuring normal physiological processes.

The *Oryza sativa* genome includes eleven *OsOSCA* genes, classified into four clades: Clade I (*OSCA1.1*, *OSCA1.2*, *OSCA1.3*, *OSCA1.4*), Clade II (*OSCA2.1*, *OSCA2.2*, *OSCA2.3*, *OSCA2.4*, *OSCA2.5*), and Clades III and IV (*OSCA3.1* and *OSCA4.1*, respectively) [47]. Not all genes are upregulated under stress; however, significant research has focused on *OsOSCA1.1*, which mediates OICI in rice roots, playing a role in stomatal closure and seedling survival under hyperosmolality [48]. OsOSCA1.4 functions as a plasma membrane Ca^2+^ channel protein, increasing cytosolic calcium levels during salt stress, which is essential for maintaining rice cell size and morphology, thus contributing to growth and osmotic stress adaptation [49].

Studies using cryo-electron microscopy (cryo-EM) have elucidated the structure and function of OsOSCA1.2, revealing a dimeric form with each subunit comprising 11 TMs and a cytosolic domain homologous to RNA recognition proteins [50]. The TM region is structurally related to the calcium-dependent ion channels and lipid flippases of the TMEM16 family. The cytosolic domain features an extended intracellular helical arm (241-266 aa) that is well-positioned to sense turgor pressure changes and transmit conformational shifts induced by membrane tension to TM6, which connects to the regulatory structure that opens the transport channel. These findings provide a framework for understanding the structural basis of osmotic concentration sensing in staple crops. In 2024, Han et al. [51] introduced a novel “lipid titration” mechanical force simulation technique using nanoscale lipid discs for “force-resolved” structural studies, capturing the conformational state of OSCA1.2 during unilateral activation. This advancement offers new insights into the molecular mechanisms of mechanosensitive channels and other membrane proteins.

## 3. Salt Stress

Salt stress significantly threatens plant growth, development, and crop yield [52]. Its effects on plants can be summarized in three main aspects: (1) Osmotic Stress: Excessive soluble salts in the soil reduce the water potential around the root surface, limiting water availability. This leads to decreased water absorption and inhibits plant growth, constituting the initial osmotic stress experienced by plants in saline environments [53]. (2) Ionic Stress: Over time, high levels of Na^+^ enter root cells through non-selective cation channels, rapidly increasing intracellular Na^+^ concentrations. This excessive sodium accumulation interferes with K^+^ absorption due to competitive inhibition, resulting in K^+^ deficiency. Elevated Na^+^ disrupts essential metabolic pathways and ionic balance, causing severe ionic stress that adversely affects plant growth [54,55]. (3) Oxidative Stress: Prolonged salt stress prevents plants from maintaining ionic balance, triggering severe oxidative stress and rapid production of ROS such as H_2_O_2_, O^2−^, and −OH. The high reactivity of ROS can damage macromolecules like lipids, nucleic acids, proteins, and carbohydrates, leading to redox imbalance and significant oxidative stress [55]. In rice, high salt stress causes morphological changes, including reduced height, root damage, leaf chlorosis, decreased biomass, lower thousand-grain weight, and reduced pollen fertility, ultimately impacting yield [56].

To cope with osmotic stress, plants accumulate osmoprotectants and lower cytosolic water potential. Organic solutes synthesized intracellularly—such as proline, betaine, choline, and organic acids along with metabolic intermediates like carbohydrates—act as effective osmotic regulators against salt stress [57]. For instance, the upregulation of trehalose biosynthesis genes, proline biosynthesis genes, and glycine betaine biosynthesis genes enhances the production of metabolites that improve rice’s salt tolerance [58,59]. In terms of ionic stress, plants have evolved mechanisms to maintain K^+^/Na^+^ homeostasis. They regulate Na^+^ absorption across the plasma and vacuolar membranes through HKTs, Na^+^/H^+^ antiporters, and NHX, minimizing ionic toxicity. Next, we will discuss two key classes of proteins involved in the response to ionic stress.

### 3.1. High-Affinity Potassium Transporters (HKT) in Rice

Members of the High-Affinity K^+^ Transporter (HKT) family play a crucial role in maintaining Na^+^/K^+^ homeostasis. Rice has the highest number of identified HKT gene family members, which are categorized into two subfamilies based on their ion selectivity: Class I and Class II. Most Class I HKT transporters are selective for Na^+^ ions, whereas Class II transporters can transport both Na^+^ and K^+^ ions. This difference is primarily due to distinct amino acid residues in the first transmembrane helix P-loop. Class I transporters have the SGGG on the first P-loop, where Ser enhances sodium ion conductance, making them specific for Na^+^. In contrast, Class II transporters possess the sequence GGGG at the same position, allowing them to select Na^+^ and/or K^+^ based on external ion concentrations.

In rice, the Class I HKT family includes five members: OsHKT1;1 to OsHKT1;5. *OsHKT1;1* is predominantly expressed in the leaf phloem. Its encoded protein, OsHKT1;1, is involved in Na^+^ transport, which can be competitively inhibited by K^+^ and Rb^+^.*OsHKT1;1* expression is positively regulated by the transcription factor OsMYBc (MYB proto-oncogene, transcription factor), which helps mitigate ionic toxicity symptoms [60]. *OsHKT1;2* is a pseudogene with no significant changes after Na^+^ and K^+^ treatment. The protein OsHKT1;3 facilitates both inward and outward Na^+^ currents, though it has weaker inward rectification [61]. Under high salinity, *OsHKT1;4* expression in the sheath inversely correlates with Na^+^ concentration in the leaves, indicating its role in Na^+^ transfer from the stem sheath to the leaves [62]. The most notable member, *OsHKT1;5*, is expressed in root and leaf xylem parenchyma cells. Its encoded protein, OsHKT1;5, functions to unload Na^+^ from the root xylem and regulate its transport to above-ground parts [63].

OsHKT2;1 is an atypical Class II transporter with a Ser residue in its first PD, giving it Na^+^ transport capabilities similar to Class I members [64]. It primarily facilitates Na^+^ absorption driven by K^+^ starvation, a process termed Na^+^ nutritional uptake. Salt-tolerant rice varieties like *Nona Boktra* and *Pokkali* express OsHKT2;2, which can co-transport Na^+^ and K^+^ when heterologously expressed in Xenopus oocytes and tobacco BY2 cells [65,66]. OsHKT2;3 may be non-functional, while OsHKT2;4 exhibits high K^+^ transport characteristics with relatively low Na^+^ transport capacity and magnesium ion transport functions, resembling MGT-type magnesium ion transport proteins [67].

Our group has been dedicated to studying the three-dimensional structure of the HKT family, aiming to elucidate the structural basis of its ion selectivity. Our group investigated the ion selectivity and transport mechanisms of the cation channels HKT2;1 and HKT2;2/1 in rice. We found that HKT2;1, due to key amino acids Ser88 and Val243, has a shorter distance (less than 3.5 Å) to the carbonyl oxygen of surrounding amino acids, facilitating Na^+^ passage. In contrast, HKT2;2/1 has Gly88 and Gly243 at the corresponding positions, with distances greater than 4 Å, making it more suitable for K^+^ transport. This study highlighted the critical sites in HKT channels that determine their Na^+^ and K^+^ ion selectivity [68]. Utilizing CRISPR/Cas9 gene editing technology, we can precisely modify these key sites by replacing specific amino acids in the ion selectivity filter to enhance rice’s tolerance to salt stress. This offers valuable insights for developing new salt-tolerant crop varieties. Additionally, Gao et al. [69] analyzed the structures of salt-tolerant and salt-sensitive mutants of OsHKT1;5, revealing structural differences and predicting stability changes in the salt-tolerant mutant protein, thereby laying the groundwork for understanding the role of OsHKT1;5 mutants in salt tolerance.

### 3.2. Mechanism of Na^+^ Transport Regulation via the SOS Pathway in Rice

Sodium ions from the soil enter root cells through HKT and other channel proteins. When intracellular Na^+^ concentrations reach a certain threshold, plants activate the Na^+^/H^+^ antiporter SOS1 in the plasma membrane via the SOS signaling pathway to respond to salt stress. First, SOS3 detects calcium signals triggered by excessive Na^+^ influx and forms a complex with SOS2, facilitating the self-activation of SOS2. This complex then translocates to the membrane through palmitoylation at the N-terminus of SOS3. Following this, SOS2 phosphorylates and activates SOS1, which extrudes excess Na^+^ from the cell, thereby enhancing salt tolerance [70]. The *sos1* loss-of-function mutant in rice shows a salt-sensitive phenotype due to excessive Na^+^ uptake in the roots and impaired Na^+^ loading in the xylem, indicating that OsSOS1 is crucial for regulating Na^+^ absorption in roots and its long-distance transport to aerial parts, aiding rice in combating salt stress.

The research investigated the molecular mechanism of SOS1 activation by analyzing its structure before and after phosphorylation [71]. Zhang et al. [71] found that each OsSOS1 monomer comprises a transmembrane domain (TMD) and three cytoplasmic domains: the helical domain, cyclic nucleotide-binding domain, and C-terminal β-roll domain. In the absence of salt stress, the helical domain interacts with TM5b, blocking the Na^+^ binding site and maintaining a self-inhibitory state.

Under salt stress, the SOS3/SOS2 complex phosphorylates SOS1, relieving its self-inhibition and inducing significant conformational changes in the intracellular regulatory domains. This disruption of the interaction between the helical domain and TM5b allows SOS1 to shift from blocking Na^+^ binding to facilitating Na^+^ transport. These findings clarify how SOS1 transitions from a resting self-inhibitory state to an active state, providing a robust structural basis for understanding its activation mechanism.

Under salt stress conditions, rice varieties display distinct defense mechanisms that contribute to their varying levels of salt tolerance. For example, *Xudao9* mitigates sodium accumulation by maintaining a low K^+^/Na^+^ ratio, while *Huageng5* alleviates sodium toxicity through the absorption of alternative ions [66]. Salt-tolerant varieties like *Nona Bokra* and *Xudao9* effectively sustain intracellular ion balance, evidenced by higher K^+^/Na^+^ ratios and enhanced uptake of beneficial ions such as K^+^ and Mg^2+^. Notably, the OsHKT2;2/1 transporter in *Nona Bokra* demonstrates strong K^+^ selectivity under low K^+^ conditions, further improving its salt tolerance [62]. These varieties also induce the expression of various salt tolerance-related genes, including those coding for ion transporters, antioxidant enzymes, and osmotic regulators [67]. For instance, *Pokkali* rapidly reduces DNA methylation levels, showcasing its adaptive flexibility in gene expression regulation [67]. In contrast, conventional varieties exhibit weaker regulatory control over these genes. The expression levels of SOS1, SOS2, and SOS3 correlate positively with salt tolerance, where SOS1 facilitates Na^+^ efflux in the root epidermis, effectively preventing sodium transport to the stem. Salt-tolerant varieties such as *Nona Bokra* and *Pokkali* show significantly higher and salt-inducible SOS1 expression compared to sensitive varieties like IR64 and IR29. The functionality of SOS1 is contingent upon phosphorylation by SOS2 and SOS3, and the robust expression of OsCIPK24/SOS2 and CBL10/SOS3 in tolerant varieties enhances Na^+^ efflux, thereby preventing Na^+^ accumulation in stems and leaves [68]. To leverage these insights for breeding strategies aimed at enhancing salt tolerance in rice, several approaches can be considered. Molecular marker-assisted selection can facilitate the rapid identification of salt-tolerant individuals by linking salt tolerance-related genes, such as OsHKT2;2/1, to specific molecular markers. Gene editing technologies, including CRISPR/Cas9, offer the potential for targeted modifications of salt tolerance genes to enhance their functionality or expression patterns. Hybridization and backcrossing can combine salt-tolerant varieties with high-yielding cultivars, resulting in new varieties that maintain both salt tolerance and desirable agronomic traits, exemplified by the potential hybridization of *Nona Bokra* with *Koshihikari* [69]. Lastly, transgenic approaches can introduce exogenous salt tolerance genes into rice, further bolstering its capacity to withstand salinity. Collectively, these strategies present a multifaceted approach that may contribute to enhancing salt tolerance in rice, which is crucial for sustaining productivity in saline environments.

## 4. Temperature Stress

As global climate change progresses, the frequency of extreme temperatures—both high and low—has increased, posing a significant challenge to agricultural development worldwide [72,73]. Rice has specific temperature requirements: the optimal temperature for germination/emergence is 27.9 °C. During the tillering stage, 28.4 °C is considered ideal for panicle differentiation. The suitable temperature for panicle initiation is 26.7 °C, while approximately 26.3 °C is optimal for anthesis [74]. Fluctuations in temperature can severely hinder rice growth and development, directly impacting yield and quality, and potentially threatening global food security. Consequently, Super hybrid rice exhibits higher yields under tropical and subtropical climate conditions but suffers damage under cold conditions [72]. Asian cultivated rice, such as *Japonica* and *Indica*, has been domesticated under specific agricultural and climatic conditions, with *japonica* showing notable adaptability to low temperatures [72,75]. When rice is exposed to temperatures outside its optimal range, it experiences abiotic stress, resulting in heat stress at high temperatures and cold stress at low temperatures.

**Figure 3:**
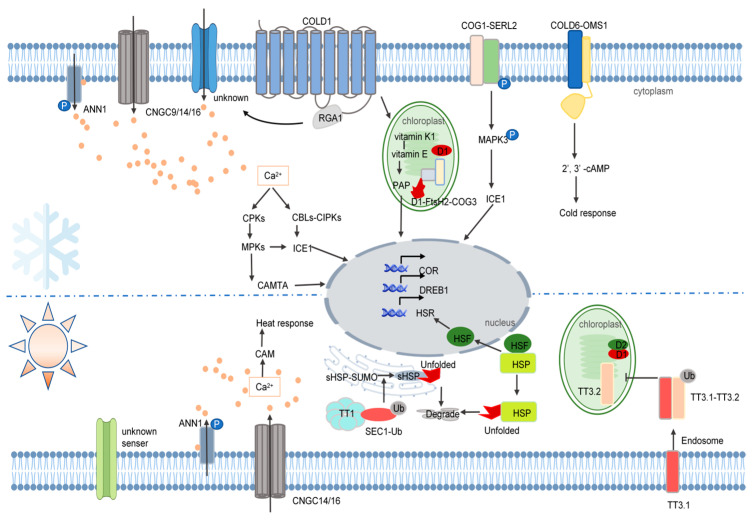
Molecular mechanisms of rice response to temperature stress. Plasma CNGC and ANN, along with unidentified calcium channels, sense temperature changes and promote the influx of intracellular Ca^2+^. This increase in calcium concentration activates downstream cascades that enhance the expression of temperature stress-responsive genes. In response to cold stress, the plasma membrane protein COLD1 enhances calcium channel activity, raising intracellular calcium levels. It also activates a vitamin K1-vitamin E signaling network in chloroplasts, leading to retrograde signaling that regulates gene expression. The protein COG1 forms a complex with SERL2, phosphorylating it to boost its activity, which activates the MPK cascade and induces the expression of COR genes. Additionally, COLD6 interacts with OMS1 to produce the second messenger 2′,3′-cAMP, initiating a series of cold response reactions. Under heat stress, the TT1 protein ubiquitinates the negative regulator SEC1, reducing the SUMOylation of sHSPs. This ensures that sHSPs effectively bind to and degrade misfolded proteins, thus enhancing heat tolerance. Concurrently, HSP proteins dissociate from HSFs, facilitating the degradation of misfolded proteins and boosting the expression of HSR genes. Following heat stress perception, TT3.1 internalizes and interacts with TT3.2, ubiquitinating it and promoting its degradation, which decreases TT3.2 levels in chloroplasts. This process enhances the structural stability of thylakoids, allowing rice to better cope with heat stress.

In response to temperature stress, rice activates various molecular mechanisms, including changes in cell wall and membrane composition and fluidity, alterations in membrane protein conformation and activity, increases in cytosolic free calcium concentrations and ROS, and adjustments in protein homeostasis [76,77]. The molecular responses to cold and heat stress share part of similar regulatory networks. Under temperature stress, membrane proteins such as OsCNGC9 (specific to cold stress), OsCNGC14, OsCNGC16, and annexins (ANNs) detect temperature signals and rapidly induce Ca^2+^ influx into the cytoplasm. This transient Ca^2+^ increase triggers downstream cascades that enhance the expression of cold-regulated or heat shock response (COR/HSR) genes [78,79,80,81]. The absence of *OsCNGC14* or *OsCNGC16* significantly diminishes or abolishes temperature stress signaling [76] (Figure 3). Furthermore, rice utilizes distinct sensors and mechanisms for cold and heat stress. Understanding these molecular mechanisms is essential for improving rice varieties, enhancing rice adaptability to temperature fluctuations, expanding rice cultivation range, and increasing grain yield. This section will detail the specific mechanisms by which rice responds to cold and heat stress.

### 4.1. Cold Stress

In low-temperature environments, the cold sensor *OsCOLD1* (*chilling-tolerance divergence 1*) gene was first identified by the Zhang team in cold-tolerant rice varieties *Japonica* [72]. *OsCOLD1* encodes a membrane-localized G protein transcription factor that senses low temperatures and activates various downstream signaling pathways. It interacts with the G protein subunit RGA1, enhancing the GTPase activity of the G protein while simultaneously activating an unidentified cold-responsive Ca^2+^ channel, which triggers Ca^2+^ influx [72]. This influx signal is transduced by calcium-dependent protein kinases (CPKs) or calcineurin B-like proteins (CBLs) and CBL-interacting protein kinases (CIPKs), leading to the activation of downstream mitogen-activated protein kinase (MAPK) cascades. These cascades induce transcription factors such as calmodulin-binding transcription activator (CAMTA) and inducer of CBF expression 1 (ICE1), which directly upregulate cold-responsive genes like dehydration responsive element binding (DREB1) and certain COR genes, enhancing rice cold tolerance [78,82,83,84]. Additionally, the Ca^2+^ influx induced by COLD1 stimulates a metabolic subnetwork involving the metabolism of vitamin E-vitamin K1 within chloroplasts. Vitamin E, as a key regulatory factor, modulates the production of the downstream metabolite 3′-phosphoadenosine 5′-phosphate (PAP), activating retrograde signaling from chloroplasts to the nucleus. This process inhibits the degradation of miRNAs and promotes the production of mature miRNAs that regulate cold tolerance [85,86,87]. However, the three-dimensional structure of COLD1 remains unresolved, and further research is needed to elucidate the conformational changes it undergoes upon cold perception and its mechanism for activating the Ca^2+^ channel.

Another gene, *COLD6*, negatively regulates rice cold tolerance and encodes membrane protein. Under normal conditions, COLD6 forms a stable complex with RGA1. During cold stress, COLD6 dissociates from RGA1, allowing RGA1 to bind to COLD1. COLD6 then forms a complex with the cold-induced membrane protein osmotin-like 1 (OSM1), which senses extracellular cold signals and triggers the accumulation of the second messenger 2′,3′-cAMP, initiating the cold defense response in rice [72,88]. Both genes contribute to the regulation of rice cold stress through different signaling pathways and may interact, though the specific molecular mechanisms require further investigation. Additionally, the membrane protein chilling tolerance in geng 1 (COG1), part of the leucine-rich repeat receptor-like proteins (LRR-RLPs), also senses low temperatures. Under cold conditions, COG1 forms a complex with the membrane receptor-like kinase OsSERL2, enhancing the phosphorylation of Ser599. This activation leads to a conformational change in the heteromeric complex, further increasing the phosphorylation of MAPK3 and activating downstream MAPK3 signaling pathways, thereby transmitting cold signals from the membrane to the cytoplasm and promoting enhanced cold tolerance in *Japonica* [83,89]. Moreover, various cellular compartments can also perceive cold stress signals. Recently, another cold-induced gene, *COG3* (*Chilling Tolerance in Geng 3*), has been identified as being expressed in both the nucleus and chloroplasts. It encodes a CAM receptor-like protein that interacts with the ATP-dependent protease filamentation temperature-sensitive H 2 (OsFtsH2) within chloroplasts, degrading the cold-damaged core subunit D1 protein of photosystem II. This degradation maintains the integrity of the D1 protein and regulates photosynthetic efficiency and cold tolerance in rice [90,91,92].

### 4.2. Heat Stress

The impact of global warming on rice is multifaceted. Under high-temperature conditions, the efficiency of photosynthesis in rice decreases, respiration increases, and pollen development and pollen tube growth are affected, leading to significant reductions in both yield and quality [93]. Photosynthesis is considered one of the most temperature-sensitive plant functions, and high temperatures reduce photosynthesis by stimulating photorespiration. The most prominent heat-labile component is the PSII (photosystem II complex) involved in photosynthetic electron transfer and ATP synthesis [93]. Under heat stress, the D1 protein of the PSII core is damaged, leading to impaired PSII function. Plant proteins play a crucial role in responding to heat stress. Extensively studied heat shock transcription factors, heat shock proteins, calcium signaling proteins, and antioxidant enzymes function through various signaling mechanisms to coordinate and enhance plant heat tolerance [94,95]. In the field of rice, the identification and study of several key heat-responsive proteins are gradually unraveling the mysteries of heat tolerance.

Lin et al. [96,97,98] have attained a succession of achievements in the study of genetic regulatory mechanisms governing rice response to high-temperature stress. Their team has successfully isolated and cloned Quantitative Trait Loci (QTLs) such as *TT1*, *TT2*, and *TT3*, which are implicated in modulating the high-temperature tolerance of rice. Notably, the identification and in-depth elucidation of the functions and mechanisms of the two regulatory genes, *TT3.1* and *TT3.2*, within the *TT3* locus have furnished a novel and profound perspective for the exploration of the molecular mechanisms underlying plant responses to extreme high temperatures. The associated research outcomes have been published in highly prestigious academic periodicals. The heat receptor identified on the cell membrane is proposed to be TT3.1. Under heat stress, TT3.1 translocates from the plasma membrane to the endosome, where it ubiquitinates the chloroplast precursor protein TT3.2, promoting its degradation in the vacuole and reducing the abundance of mature TT3.2. This process protects thylakoids and safeguards chloroplasts from heat-induced damage [97]. However, the mechanism by which TT3.2 accumulation leads to chloroplast damage remains unclear. The translocation of TT3.1 is essential for relaying heat stress signals from the cell surface to intracellular organelles [97]. Research teams have discovered heat tolerance mechanisms through large-scale individual screening and heat tolerance phenotype identification, providing evidence that breeding can improve rice heat tolerance. In addition to the laboratory screening and identification mentioned above, we can also enhance rice heat tolerance by introducing heat-tolerant genes.

Heat stress also causes protein denaturation in rice. During this stress, heat shock proteins (HSPs) dissociate from heat stress transcription factors (HSFs). HSPs function as molecular chaperones, binding to and degrading misfolded proteins to maintain homeostasis, while HSFs activate the expression of downstream heat-responsive genes [99]. This HSF/HSP pathway is a well-established response mechanism to heat stress. Rice TT1 enhances heat tolerance by influencing HSF protein abundance. *TT1* encodes the α2 subunit of the 26S proteasome, which degrades ubiquitinated proteins, effectively removing toxic denatured proteins and supporting the heat response [100]. The small ubiquitin-like modifier (SUMO)-conjugating enzyme 1 (SCE1) plays a crucial role in the TT1-mediated heat stress response. *SCE1* encodes a SUMO E2 conjugase that regulates the abundance and SUMOylation of small heat shock proteins (sHSPs), such as HSP24.1, thereby influencing protein folding. Consequently, SCE1 acts as a negative regulator of heat tolerance in rice. SCE1 interacts with TT1, and under high-temperature stress, TT1 promotes the ubiquitination of SCE1, targeting it for degradation by the 26S proteasome. This reduces SCE1 protein levels, thereby enhancing rice heat tolerance [98].

## 5. Heavy Metal Stress

With the advancement of agriculture and industry, heavy metal pollution in soil is escalating, particularly in irrigated agricultural areas where concentrations of cadmium (Cd), mercury (Hg), chromium (Cr), lead (Pb), and arsenic (As) have significantly increased. This contamination adversely affects plant growth and development, posing threats to future food security and human health [101]. Rice, in particular, is highly susceptible to the uptake and accumulation of heavy metals like Cd and As from the soil, leading to elevated levels in grains. Cd inhibits the absorption of essential nutrients such as zinc (Zn), iron (Fe), and magnesium (Mg), thereby reducing plant biomass [102]. Cd is absorbed by rice roots through irrigation water and soil via transport proteins and is subsequently transported throughout the plant, affecting all growth stages. Due to its high bioavailability and environmental persistence, Cd represents the greatest threat to rice cultivation. Understanding the molecular mechanisms by which rice responds to Cd stress is crucial for enhancing stress resistance in this important crop.

The response of rice to Cd stress can be divided into several stages. The first stage involves the excessive uptake of Cd ions from the soil into the roots, facilitated by membrane proteins. Once in the cytoplasm, the second stage includes Cd ions being captured by high-affinity metal-binding proteins that block further detrimental effects, or they are transported into organelles for storage and isolation. The third stage includes the upward transport of excess Cd through the xylem from roots to shoots and grains, ultimately impacting food safety [103]. This section focuses on the molecular mechanisms of Cd^2+^ transport and metal chelation in rice. Understanding the response of rice to metal stress at the protein level can provide more precise insights for protein modification, thereby laying a theoretical foundation for subsequent gene editing and breeding efforts.

Controlling Cd^2+^ transport is the first step in rice response to Cd stress. Cd^2+^ transport proteins on rice cell membranes often compete with essential divalent metal trace elements such as manganese (Mn), Zn, and Fe. The Natural resistance-associated macrophage protein (Nramp) family, which typically consists of 11 or 12 transmembrane domains, plays a crucial role in binding metal ions, particularly Cd. Structural analyses show that Nramp can bind metal ions in an outward-open conformation, releasing them into the cytoplasm [104,105]. OsNramp1 and OsNramp5 are primary Cd^2+^ transport proteins located in the outer and inner cortical layers of rice roots, respectively, and they also competitively absorb Mn from the soil [106,107,108]. Knockout of *OsNramp1* results in lower levels of toxic Cd^2+^ in mutant leaves and grains, although it also leads to a significant decrease in Mn^2+^ levels [108]. Jian et al. initially validated that the protein encoded by *Nramp5* is capable of transporting cadmium ions, thereby furnishing a prospective target for diminishing the cadmium content within crops via gene selection and modification [109]. In the rice variety *Pokkali*, the gene *OsNramp5* responds to Cd stress by increasing its copy number, which enhances OsNramp5 protein expression and improves the absorption of both Mn^2+^ and Cd^2+^ in root cells [106]. After entering the cells, most Cd^2+^ is transported to the vacuole for storage by the Cadmium/zinc-transporting ATPase 3 (OsHMA3), which has a strong metal-binding capacity due to its cysteine repeat sequences [110]. Most Mn^2+^ is transported to the xylem by the metal tolerance protein 9 (OsMTP9), which competes with Cd^2+^ for transporters on the cell membrane, resulting in reduced Cd^2+^ levels in the xylem and lower Cd^2+^ content in rice grains under stress [106,107].

Additionally, the root epidermal cell membrane takes up Cd through the cadmium transporter (OsCd1), a member of the major facilitator superfamily (MFS). A mutation from aspartic acid (in *Indica*) to valine (in *Japonica*) at position 449 significantly reduces OsCd1 transport capacity, decreasing Cd^2+^ accumulation in *Japonica* [111].

In response to Cd stress, controlling Cd^2+^ absorption and reducing its transport to grains is crucial, as is the function of transport proteins that extrude Cd^2+^. Cd stress induces the transcriptional upregulation of genes from the zinc-regulated transporter, iron-regulated transporter-like protein (ZIP) family [112]. While the structure of rice ZIP proteins is not fully elucidated, they are known to have multiple conserved metal ion-binding sites [113,114]. OsZIP1 is highly expressed in rice roots and functions as a metal detoxification transporter. Under normal conditions, OsZIP1 is expressed at low levels, ensuring the loss of divalent metal ions required for growth. When Cd stress occurs, increased Cd^2+^ levels induce OsZIP1 overexpression, lowering Cd^2+^ concentrations and preventing excessive accumulation [112,115].

Heavy metal ions like Cd can enter cells and lead to the accumulation of ROS, which interact with proteins and lipids, resulting in decreased enzyme activity. To counteract Cd stress, rice employs high-affinity metal chelators to bind Cd^2+^, thereby reducing excess ROS and mitigating Cd-induced damage. Metal-binding proteins such as MTs and OsPCS play significant roles in this response [102]. MTs are cysteine-rich proteins expressed throughout rice growth stages, and their transcription is upregulated by metal ions [116]. They bind metal ions through thiol groups, forming non-toxic complexes that reduce toxicity [117]. X-ray crystallography studies have revealed the metal-binding sites and coordination geometry of MTs, which typically consist of two independent domains that prevent cellular damage [118].

Under Cd stress, OsPCS rapidly induces the synthesis of phytochelatin (PC), a protein rich in thiol groups that can bind heavy metal ions to form non-toxic complexes. These complexes are transported to the vacuole by the ABC transporter OsABCC1, reducing free heavy metal content and minimizing toxicity [119]. Disruption of OsPCS1 significantly increases rice sensitivity to As and Cd stress [117]. Furthermore, antioxidant enzymes (such as GPX) play essential roles in alleviating heavy metal-induced oxidative damage and scavenging ROS. Under stress, rice root tips generate and transmit ROS signals, with OsRBOH, located on the plasma membrane, being transcriptionally upregulated. This regulation occurs through the RBOH–ROS–Auxin signaling pathway, modulating auxin responses and cell wall remodeling, ultimately reshaping root architecture to mitigate uneven distributions of heavy metal stress.

## 6. Different Stresses Share Common Response Mechanisms

Rice exhibits a series of complex response mechanisms when facing various abiotic stresses, and these mechanisms share commonalities across different stress conditions. These common response mechanisms not only help rice survive under adverse conditions but also provide an important theoretical basis for breeding stress-resistant varieties. (1) Regulation of Redox Balance: Reactive oxygen species (ROS) levels typically increase under various abiotic stresses, but their specific response mechanisms vary depending on the type of stress [120,121]. Under drought conditions, ROS induce stomatal closure by activating the MAPK cascade and ABA signaling pathway, reducing water loss. Under salt stress, ROS regulate ion balance and the accumulation of osmoprotectants through the calcium signaling pathway and MAPK cascade. Under high-temperature stress, ROS induce the expression of heat shock proteins (HSPs) to protect protein structure and function [122]. Under low-temperature stress, ROS activate the expression of cold-responsive genes through the calcium signaling pathway and MAPK cascade, enhancing cold tolerance [72]. Under heavy metal stress, ROS activate the antioxidant enzyme system to scavenge excess ROS while inducing the expression of metal-chelating proteins to mitigate heavy metal toxicity [123]. (2) Activation of ABA Signaling Pathway: A major phytohormone, abscisic acid (ABA), plays an essential part in acting toward varied range of stresses like heavy metal stress, drought, thermal or heat stress, high levels of salinity, low temperature, and radiation stress [124]. ABA induces stomatal closure by binding to ABA receptors, activating SnRK2 kinases, and phosphorylating SLAC1 and QUAC1 ion channels, leading to anion and K⁺ efflux. This process is a critical mechanism for plants to cope with drought and salt stress [32]. Additionally, ABA acts by modifying the expression level of genes and subsequent analysis of cis- and trans-acting regulatory elements of responsive promoters. Under temperature stress, ABA helps plants cope with high temperatures by regulating the expression of the *HSP70* gene [122]. Under heavy metal stress, ABA alleviates heavy metal toxicity by regulating the expression of the *Metallothionein 2 (MT2)* gene [123]. (3) Accumulation of Osmoprotectants: Under various stress conditions, rice accumulates osmoprotectants such as proline, soluble sugars, and glycine betaine to maintain cellular osmotic balance and protect cellular structures [125,126]. These substances play a key role in drought, salinity, and temperature stresses. For example, the accumulation of proline can stabilize proteins and cell membranes, while soluble sugars act as osmoprotectants and energy sources, and glycine betaine protects enzyme activity and membrane structure. (4) Activation of Calcium Ion (Ca^2+^) Signaling Pathway: Calcium ions act as second messengers involved in the transduction of multiple stress signals [127]. Calcium-dependent protein kinases (CDPKs) and calmodulin (CaM) are activated, regulating downstream stress responses. This mechanism is particularly important in cold and heat stresses [128]. For example, under cold stress, OsCOLD1 activates calcium channels, increasing intracellular calcium ion concentrations, which in turn activate downstream MAPK cascades and induce the expression of cold-responsive genes [72]. (5) Activation of MAPK Cascade Pathway: The MAPK cascade is a core stress signal transduction pathway that passes stress signals through the MAPKKK-MAPKK-MAPK cascade and regulates gene expression via transcription factors such as WRKY and MYB [129]. This mechanism plays a crucial role in drought, salinity, temperature, and heavy metal stresses. For example, under heat stress, TT1 ubiquitinates SCE1, reducing the SUMOylation of sHSPs, ensuring that sHSPs effectively bind to and degrade misfolded proteins, enhancing heat tolerance. (6) Expression of Stress-Related Genes: Under multiple stress conditions, rice induces the expression of similar stress-related genes, such as LEA proteins, heat shock proteins (HSPs), and antioxidant enzyme genes. These genes enhance cellular protection. For example, LEA proteins protect cellular structure and function [130], HSPs assist in the proper folding and repair of proteins, and antioxidant enzyme genes enhance ROS scavenging capacity [131].

These common response mechanisms form a complex regulatory network that enables rice to survive and adapt under various abiotic stress conditions. ROS, as upstream signaling molecules, activate ABA, Ca^2+^, and the MAPK cascade. ABA and Ca^2+^ transmit stress signals by regulating the MAPK cascade and the expression of stress-responsive genes. The MAPK cascade, in turn, modulates the activity of transcription factors, inducing the expression of stress-related genes. Ultimately, the accumulation of osmoprotectants and the expression of stress-related genes enable plants to adapt to and cope with stress conditions.These mechanisms not only provide a theoretical basis for understanding the stress tolerance of rice but also offer scientific support for breeding stress-resistant varieties through targeted breeding and genetic engineering approaches.

## 7. Conclusions

Abiotic stress, exacerbated by environmental changes such as climate shifts and soil degradation, poses significant challenges to rice cultivation, complicating its normal growth and increasing susceptibility to diseases. Rice often encounters multiple environmental pressures simultaneously, making its response to abiotic stresses like drought, salinity, extreme temperatures, and heavy metal ions highly complex and interrelated. This response involves a coordinated regulation of numerous proteins, rather than individual ones, through intricate feedback mechanisms to ensure normal growth and development.The genetic improvement of rice primarily targets tolerance to moderate stress conditions, such as moderate drought and saline-alkali soils, which are frequently encountered in actual cultivation and directly impact rice growth and yield [132,133]. Through traditional breeding and modern biotechnology, researchers are working to develop rice varieties that can adapt to these environmental pressures, thereby enhancing the stability and sustainability of agriculture. However, with global climate change and population growth, future research should also focus on tolerance under extreme stress conditions to ensure the long-term safety of rice production and the stability of the food supply.

Recent advances in structural biology have enhanced our understanding of the regulatory and signaling pathways involved in rice’s stress response. For instance, the detailed elucidation of the SOS3 signaling pathway under salt stress provides insights into the molecular mechanisms by which rice adapts to such conditions, offering precise targets for breeding stress-resistant varieties. However, many stress response proteins remain unidentified, and the operational mechanisms of known proteins are not fully understood, posing challenges to a comprehensive understanding of rice’s stress response at the molecular level.

Historically, research on plant responses to abiotic stress has predominantly focused on the model plant *Arabidopsis*, leaving gaps in our understanding of rice-specific responses. However, rice has evolved unique protein response mechanisms and signaling pathways to adapt to its distinct growth environment and stress conditions. For example, the core components of the ABA signal in rice are conserved with *Arabidopsis*, but there are differences in the regulation and function of these proteins. For instance, rice has a larger family of SnRK2 kinases (ten members) compared to Arabidopsis (three members), and some rice SnRK2s exhibit unique phosphorylation targets and stress-specific activation patterns [134]. This suggests that rice may have evolved more specialized roles for SnRK2 kinases in response to abiotic stresses such as drought and salinity. Additionally, there are functionally specific stress-responsive genes in the rice genome, such as the salt-tolerant gene NHX1 [135], which function differently in *Arabidopsis* related to plant growth and development, root development, and stress resistance [136]. Therefore, it is crucial to investigate whether rice has evolved distinct protein response mechanisms and signaling pathways. Future research should aim to identify stress response proteins in rice, analyze their structures, and employ targeted mutagenesis to unravel the specific signaling mechanisms involved. With the advancement of technology, integrating multi-omics data, including genomics, transcriptomics, and proteomics, can accelerate the identification of resistance genes, analyze key responsive genes and signaling pathways, and elucidate the roles of different proteins in rice resistance. This approach helps identify targets for improving rice resistance. Additionally, utilizing genetic technologies such as gene editing to precisely modify resistance genes, combined with traditional breeding methods, can achieve precise breeding and effectively enhance rice resistance, optimizing the balance between stress responses and growth.

In conclusion, this review focuses on the protein level to review the different response mechanisms of rice to common abiotic stresses. It summarizes the functions and regulatory networks of key proteins under various stress conditions, deepening the comprehensive understanding of rice stress tolerance mechanisms. This work provides scientific targets for subsequent gene engineering and precision breeding and offers theoretical support for improving rice quality and yield.
